# Ultrasound as a Tool to Assess Body Fat

**DOI:** 10.1155/2013/280713

**Published:** 2013-08-26

**Authors:** Dale R. Wagner

**Affiliations:** Human Movement Science Program, Health, Physical Education and Recreation Department, Utah State University, 7000 Old Main Hill, Logan, UT 84322-7000, USA

## Abstract

Ultrasound has been used effectively to assess body fat for nearly 5 decades, yet this method is not known as well as many other body composition techniques. The purpose of this review is to explain the technical principles of the ultrasound method, explain the procedures for taking a measurement and interpreting the results, evaluate the reliability and validity of this method for measuring subcutaneous and visceral adipose tissue, highlight the advantages and limitations of ultrasound relative to other body composition methods, consider its utility to clinical populations, and introduce new body composition-specific ultrasound technology. The focus of this review is adipose, although various tissue thicknesses (e.g., muscle and bone) can be measured with ultrasound. Being a portable imaging device that is capable of making fast regional estimates of body composition, ultrasound is an attractive assessment tool in instances when other methods are limited. Furthermore, much of the research suggests that it is reliable, reproducible, and accurate. The biggest limitations appear to be a lack of standardization for the measurement technique and results that are highly dependent on operator proficiency. New ultrasound devices and accompanying software designed specifically for the purpose of body composition assessment might help to minimize these limitations.

## 1. Introduction

 An accurate assessment of body composition is important to identify health risk associated with excessively high or low amounts of body fat, monitor changes in body composition associated with certain diseases, as an aid to developing weight loss or weight gain programs and assessing the effectiveness of nutrition and exercise interventions, and to monitor age-related changes in body composition. Many different methods for the assessment of body composition exist. Some methods are referred to as laboratory methods because they are typically available in only clinical and research settings. Common laboratory methods include dual-energy X-ray absorptiometry (DXA), densitometry obtained from underwater weighing or air displacement plethysmography, and hydrometry from isotope dilution. Other methods, such as skinfolds, bioelectrical impedance (BIA), anthropometric measurements, and weight : height indices, involve minimal and easily portable equipment; thus, they are more practical for the practitioner and categorized as field methods. Numerous review articles detailing these methods and techniques have been published. Ultrasound can also be used for body composition assessment; however, even the most popular and widely-cited body composition assessment texts [1–3] and review articles [4–11] give only a cursory mention, at best, of ultrasound.

 Most people are familiar with the biomedical diagnostic application of ultrasound, including the visualization of a fetus during a prenatal exam. Less well known is the use of ultrasound to measure fat and muscle thicknesses in humans. The use of ultrasound to measure fat thickness in humans dates back to the mid-1960s [12, 13]. Despite a nearly 50-year history of ultrasound being used to measure subcutaneous adipose tissue, this technology seems to be used far less than the previously mentioned methods for body composition assessment, and many students, researchers, and clinicians are not familiar with its usefulness and versatility as a body composition assessment tool. Thus, the purpose of this review of ultrasound technology is to explain the technical principles of the method and measurement procedures, evaluate the reliability and validity, address advantages and limitations relative to other methods, consider its various applications to different populations, and examine new body composition-specific ultrasound technology. This is not an all-inclusive review of the vast amount ultrasound literature available; however, several online databases, including PubMed, Scopus, and Google Scholar, were searched to ensure the most useful, and pertinent publications were included in this review.

## 2. Technical Principles

 Sound travels in the form of a cyclical wave, and humans can detect sound with a frequency in the range of about 20 to 20,000 Hz. Ultrasound operates at a frequency >20 KHz, and frequencies >2 MHz are used for ultrasonic imaging. Piezoelectric crystals in the transducer of the scan head produce pulses of ultrasound [14]. The ultrasound beam is transmitted through the skin. When the beam comes in contact with a tissue interface (e.g., skin-subcutaneous fat, fat muscle, and muscle bone), it is partially reflected back to the transducer as an echo. Thus, the transducer has a dual function of transmitting the ultrasound and receiving it. The echoes are converted into signals for processing by the transducer. The strength of each reflected wave is represented by a dot, and the position of the dot represents the depth from which the echo was received. The dots are combined to form an image.

**Figure 1:**
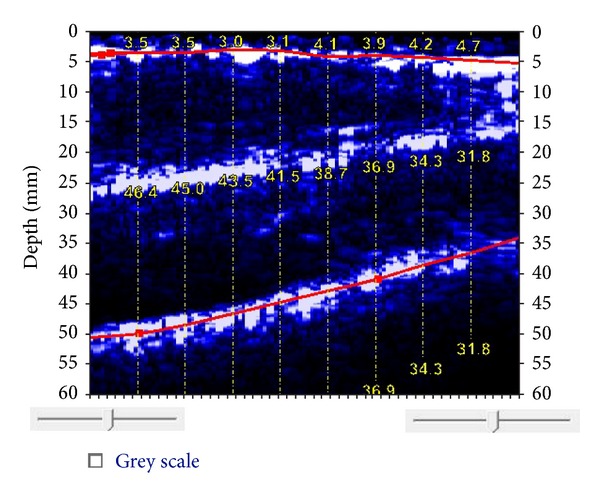
BodyMetrix ultrasound image of thigh. The top line indicates the subcutaneous fat-muscle interface (average thickness of 3.69 mm). The bottom line indicates the muscle-bone boundary. The muscle thickness ranges from 32.0 mm to 46.6 mm. The white layer in the center is the boundary of the rectus femoris and vastus intermedius.

The fundamental principle of ultrasound imaging is reflection of ultrasound waves from tissue in the path of the beam. The amount of sound reflected is dependent on the changes in acoustic impedance between two tissue interfaces. Acoustic impedance is the product of tissue density and acoustic velocity [15]. Air has almost no impedance, while fat and muscle have impedances of 0.138 g·cm^−1^·s^−1^ and 0.170 g·cm^−1^·s^−1^, respectively, and bone has a relatively high impedance of 0.78 g·cm^−1^·s^−1^. Homogenous zones with relatively uniform acoustic impedance are free of echoes. Because the acoustic impedances of fat and muscle are similar, there is a weaker echo for the fat-muscle interface than for the muscle-bone interface. For example, the software for a relatively new portable ultrasound that converts ultrasound images to body fat percentages (Body View software, IntelaMetrix, Inc., Livermore, CA) assumes an acoustic reflection coefficient of 0.012 for the fat-muscle interface, but 0.22 for the muscle-bone interface [16]. The relative strength, or amplitude, of echoes is represented by the brightness of the image on the computer screen. Strong reflections appear white; weaker reflections appear grey, and no echoes are black. This produces a two-dimensional grey-scale image with white borders for the skin-subcutaneous fat and muscle-bone interfaces and a visible, but less distinct, border for the fat-muscle interface (Figure 1).

Ultrasound transducers vary with regard to mode and frequency. An A-mode, or amplitude-mode, transducer relies on a narrow beam to scan tissue discontinuity and produces a spike on a graph. B-mode, or brightness modulation, scanning uses a linear array to produce a two-dimensional image by combining A-mode signals from various directions [17]. The higher the ultrasound frequency the greater the resolution, but there is a decrease in penetration. There is not yet any standardized protocol for using ultrasound to measure subcutaneous fat, but the majority of the studies included in this review have used a 5 MHz, B-mode transducer; however, a relatively new portable ultrasound device (BodyMetrix, BX2000, IntelaMetrix, Inc., Livermore, CA) designed and marketed specifically for body composition assessment uses a 2.5 MHz, A-mode transducer, and Pineau and colleagues [18, 19] validated body fat measurements against DXA using an A-mode transducer with a 2.25 MHz frequency.

## 3. Measurement and Interpretation

**Figure 2:**
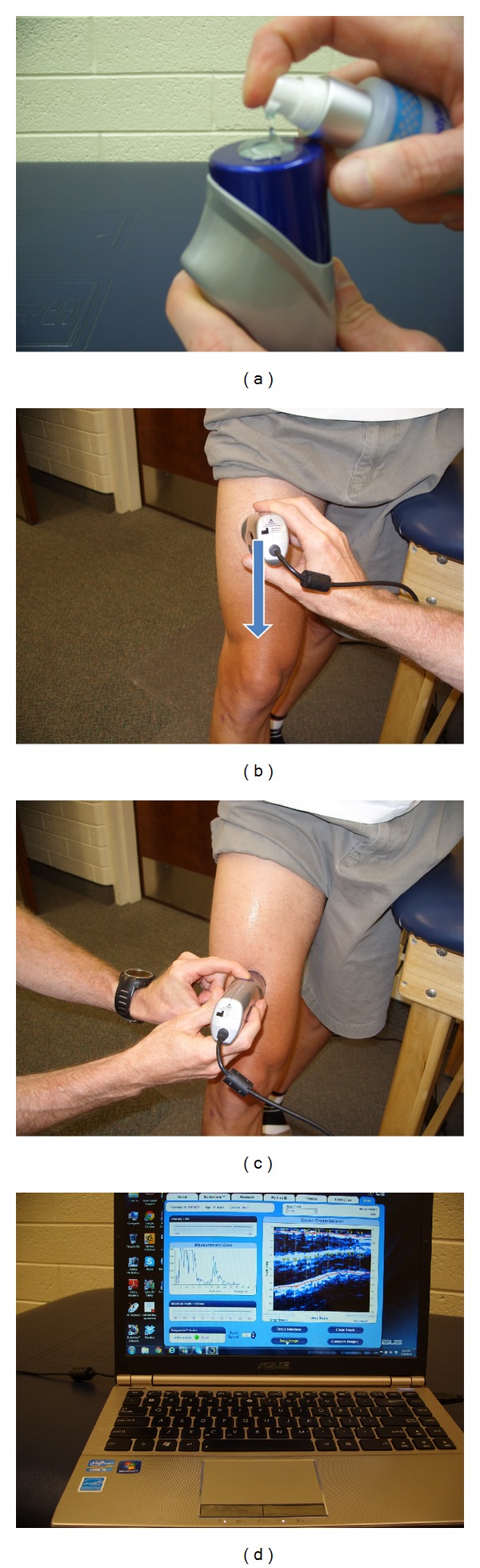
Measurement sequence for an ultrasound image scan of the thigh. (a) Gel applied to the ultrasound head for lubrication. (b) Beginning of scan. (c) End of scan. (d) Scanned image appears on screen and can be saved for future analysis.

 The procedure for ultrasound scanning is simple. Gel is placed on the head of the transducer and/or the skin at the site to be measured. This creates a close bond between the transducer and skin reducing artifact and making it easier to move the transducer over the skin. With the ultrasound on, the transducer is slid across the measurement site without loss of contact with the skin. The amount of movement depends on the purpose of the test; for a single site comparable to a skinfold, the movement is only about ±5 mm, but an entire region (e.g., thigh) can be scanned if that is the objective. A scan takes only a few seconds. Once scanned, the image on the monitor can be saved for analysis.  Figure 2 illustrates the measurement sequence.

Although the scan is a simple procedure, the interpretation is more difficult and subjective. Interfaces (skin-fat, fat-muscle, and muscle-bone) appear as continuous bands of bright light (Figure 1). However, light streaks representing fascia could be misinterpreted as an interface. The tester must be able to identify interfaces, particularly the adipose-muscle interface, and accurately measure the tissue layer of interest. Tissue thickness measurement is accomplished with electronic calipers. Identification and placement of the two caliper points defining the boundaries of the tissue to be measured requires practice to improve the objectivity of the measurement. Interpretation of ultrasound images is thought to improve with experience [12, 20, 21]. However, Inoue and colleagues [22] reported on an automated discrimination method for identifying tissue boundaries using a novel, portable ultrasound called Ubiquitous Echo. The automated method was compared to manual discrimination by an experienced observer in 11 subjects at 3 different anatomical sites. The automated method had a high discrimination rate of about 80%, and they concluded that there were relatively small discrimination errors. Unfortunately, there are not any more recent publications on Ubiquitous Echo, and although it is purported to be commercially available [22], the device and software could not be located using Internet search engines.

 Currently, there are no universally accepted guidelines for measuring subcutaneous adipose tissue with ultrasound. Toomey and colleagues [23] recently examined the technical aspects of using ultrasound to measure subcutaneous adipose tissue. They reported that when the operator applied maximal force to the transducer, subcutaneous adipose tissue thickness was reduced by 25–37% depending on the site of measurement. However, no significant difference was found when sites were scanned longitudinally versus vertically. They provided some recommendations, but there is still a lack of standardization with regard to several aspects of ultrasound measurement (e.g., optimal scanning frequency and distance or length of scan, etc.).

## 4. Reliability and Validity 

### 4.1. Subcutaneous Adipose Tissue

 Nearly 50 years ago, researchers reported strong correlations between ultrasound measurements of subcutaneous fat and measurements made by needle puncture (*r* = 0.98) [13] and electrical conductivity (*r* = 0.98) [12]. Several researchers reported that ultrasound was an acceptable alternative to radiography for measuring tissue thicknesses [13, 24]. Reliability was also reported as excellent (*r* > 0.985) [13, 25]; however, Borkan and colleagues [26] reported that the intraobserver reliability of skinfolds was better than that of ultrasound at almost every one of 15 sites measured. From the late 1960s through the 1980s numerous investigators reported significant correlations between ultrasound and skinfold caliper measurements taken at various anatomical locations [12, 13, 24–27]; however, there was agreement among several researchers that the strength of the correlation varies considerably by site and gender [25–28].

 During this early period of investigating the validity of ultrasound from the late 1960s through mid-1980s, there were different opinions as to which method, ultrasound or skinfold, best measured subcutaneous fat. In a 1967 study, Sloan [29] compared seven skinfold sites to ultrasound measures taken at the same locations and to densitometry from underwater weighing. Sloan reported similar, yet slightly greater, correlations for each skinfold site and body density than for the corresponding ultrasound measurement and body density. The accuracy of a body density prediction from the ultrasound measurements was also below the accuracy of a prediction from skinfolds. In contrast, Hawes et al. [24] reported stronger correlations between ultrasound and radiography at the iliac crest (*r* = 0.97) and greater trochanter (*r* = 0.83) than for skinfold and radiography at the same sites (*r* = 0.82 and 0.47, resp.). When using 4 common skinfold sites, Haymes et al. [28] noted that the reproducibility of the ultrasound values (*r* = 0.87 to 0.98) were marginally lower than caliper measurements (*r* = 0.98 to 0.99). Borkan et al. [26] measured 15 sites with calipers and ultrasound. Skinfold correlated better with fat weight, as measured by potassium-40 counting, than did ultrasound, and they concluded that skinfolds were a better measure of subcutaneous fat than ultrasound. However, in a landmark study by Fanelli and Kuczmarski [27], it was suggested that ultrasound was equal to skinfolds for predicting body fat. These researchers measured 7 sites on 124 men ranging in body fat from 3.5% to 32.7%. Hydrodensitometry was the criterion method. The correlation coefficients between body density and skinfolds and body density and ultrasound were similar, with skinfolds performing marginally better. But, the prediction equation for body density from ultrasound (*r* = 0.809, SEE = 0.0078 g/cc) was slightly superior to that using skinfolds (*r* = 0.779; SEE = 0.0083 g/cc). Subsequently, this research team developed a body density prediction equation from ultrasound measurements of 44 obese adults [30]. The regression equation from ultrasound (*r* = 0.819, SEE = 0.0095 g/cc) was superior to the prediction from skinfold calipers (*r* = 0.690, SEE = 0.0125 g/cc).

 In 2012, Leahy et al. [31] took ultrasound and DXA measurements in 83 men and 52 women, aged 18–29 years. They found that a single ultrasound measure of subcutaneous adipose tissue at the abdomen was highly correlated with body fat percentage in both men (*r* = 0.907) and women (*r* = 0.905). They added a lower limb measurement to develop a body fat percentage equation for men (abdomen + thigh; *r* = 0.947, SEE = 1.9%) and women (abdomen + calf; *r* = 0.909, SEE = 3.0%) with good predictive accuracy. In addition to these body fat percentage equations of Leahy et al. [31], others have used ultrasound to predict the body density of lean men [27], lean women [32], obese adults [30], Japanese men and women [33], and sumo wrestlers [34], the body fat percentage of physically active British and Chinese men [35], and the fat mass of prepubertal Japanese children [36].

### 4.2. Visceral Adipose Tissue

In 1990, Armellini and colleagues [37] introduced an ultrasonographic technique to measure intra-abdominal thickness. Their procedure correlated well with computed tomography (CT) (*r* = 0.669, *P* < 0.001). Subsequent studies by this research team validated that ultrasound was able to measure small reductions in intra-abdominal fat [38] and that intra-abdominal thickness measured by ultrasound was the most powerful predictor of visceral adipose tissue area [39]. Meanwhile, Suzuki et al. [40] used ultrasound to develop the abdominal wall fat index, which was the ratio of the maximum thickness of preperitoneal fat to the minimum thickness of subcutaneous fat. This index was closely correlated to the ratio of visceral fat to subcutaneous fat obtained by CT (*r* = 0.746, *P* < 0.0001). Abe and colleagues [41, 42] indirectly estimated deep adipose tissue by subtracting the subcutaneous fat, which was assessed by ultrasound at various body segments, from total body fat. They reported significant strong correlations (*r* = 0.79–0.95) between the segmental subcutaneous adipose tissue volumes estimated by ultrasound and observations by magnetic resonance imaging (MRI). These studies in the 1990s were revolutionary in establishing ultrasound as an alternative to the more costly and sophisticated imaging techniques of CT and MRI for assessing the different layers of fat rather than just fat versus nonfat tissue. 

**Figure 3:**
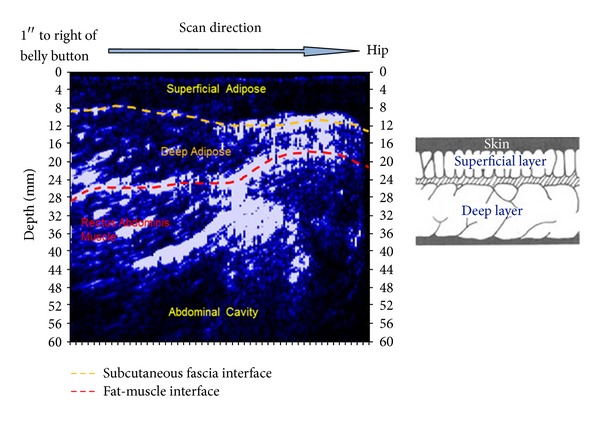
Image and illustration of an ultrasound scan showing superficial adipose tissue and deep adipose tissue (provided with permission from IntelaMetrix, Inc., Livermore, CA).

Much of the current research in body composition focuses on partitioning adipose tissue because cardiometabolic risks are associated more with visceral adipose tissue than subcutaneous adipose tissue, and the ratio between visceral and subcutaneous fat is critical in predicting this risk [43]. Recent research suggests that ultrasound is a reliable, valid, and fast method for assessing both subcutaneous and visceral adipose compartments. Using Lin's concordance correlation (*ρ*), Bazzocchi et al. [44] reported strong relationships between CT and ultrasound measures of visceral and subcutaneous parameters (*ρ* = 0.85–0.96), excellent intraobserver and interobserver agreement (ICC = 0.90–0.99), and fast ultrasound scan times of 95 ± 21 s for lean subjects and 129 ± 33 s for obese subjects. However, Shuster et al. [45] caution that the reliability and accuracy of ultrasound for assessing visceral adiposity are highly dependent on operator skill.  Figure 3 shows an example of an ultrasound image with superficial and deep adipose tissue identified. This scan was taken approximately 1 inch (2.5 cm) to the right of the umbilicus to within 3 inches (8 cm) of the iliac bone. For details regarding ultrasound as a diagnostic tool for assessing visceral adipose tissue and ultrasound techniques to measure different compartments of visceral adipose tissue refer to the literature reviews of Iacobellis [46] and Vlachos et al. [47].

### 4.3. Other Tissues

The present review is limited to using ultrasound for the assessment of body fat. However, one of the attractive features of ultrasound as a body composition tool is its ability to measure the thickness of other tissues as well such as muscle and bone. For information regarding the reliability and validity of ultrasound for measuring the thicknesses of muscle and bone the reader is directed to the work of Mayans et al. [48] and Karjalainen et al. [49], respectively.

## 5. Advantages and Limitations

Advantages and limitations of ultrasound for assessing body fat are summarized in Table 1. In a recent review of body composition assessment for athletes, ultrasound was classified as a laboratory method [50]. However, given its small size (e.g., BodyMetrix ultrasound wand is 6.5 × 2 inches and 8 oz.) and portability, ultrasound is also a viable field method. This is a tremendous advantage over other large, immobile, laboratory imaging methods, such as DXA, CT, and MRI. Also, there is no ionizing radiation with ultrasound as there is for DXA and CT. Ultrasound is safe and does not present a detectable health risk. At the levels used for biomedical purposes, ultrasound does not heat the body beyond the normal physiological range [15]. Other laboratory methods such as hydrostatic weighing and air displacement plethysmography are limited to whole-body assessment of body density. In contrast, ultrasound can provide a site-specific evaluation of skin, adipose, and muscle thicknesses. Increasingly, it is being used to discriminate visceral from subcutaneous adipose tissue [44]. Additionally, the ultrasound procedure is faster than other laboratory procedures. Furthermore, ultrasound is far less costly than other laboratory methods.

Although ultrasound is more expensive than skinfold calipers or hand-held BIA devices, it offers several advantages over these field methods. Skinfolds measure fold thickness rather than tissue thickness, and this method is not recommended for assessing obese or elderly individuals [2]. BIA values can vary based on hydration status; thus, pretesting hydration guidelines are recommended [2]. In contrast, ultrasound is not limited by subcutaneous fat thickness, loose connective tissue, or hydration status. Unlike skinfold calipers, when applied correctly there is almost no tissue compression with ultrasound. Furthermore, ultrasound can measure muscle thickness, as well as differentiate subcutaneous adipose tissue from visceral adipose tissue, limitations of other field techniques.

 Despite the many advantages of ultrasound over other methods, there are several limitations. First, some artifact is inherent in the ultrasound method. For example, fascia could be mistaken for the boundary layer between subcutaneous fat and muscle. Additionally, pressing the transducer onto the client's skin with too much force will significantly reduce the subcutaneous adipose tissue thickness [23]. Thus, considerable skill, training, and practice are necessary to produce reliable and valid results. Another limitation is that the procedures for using ultrasound for the purpose of body composition are not as clearly defined or standardized as they are with other body composition methods. For example, text books have been published that detail anatomical placement, measurement technique, and pretesting guidelines for anthropometry, skinfolds, and BIA [2, 51], yet there is considerable variability in the body composition literature for ultrasound frequencies and measurement sites. Toomey et al. [23] recently made technical recommendations for using ultrasound to measure subcutaneous adipose tissue, but much more detail and standardization is needed.

## 6. Applications to Special Populations

 The unique features and characteristics of ultrasound make it a valuable tool in the assessment of body composition of certain clinical populations where other body composition methods have failed or are severely limited. For example, examiners are advised against using the skinfold method to estimate the body composition of obese individuals because of greater variation in the depth at which the caliper tips can be placed, more variability in the compressibility of adipose tissue in obese clients, and reduced interrater reliability [2]. Skinfold thicknesses and anthropometric indices such as waist-to-hip ratio have poor validity in evaluating the intra-abdominal fat of obese children [52]. In contrast, ultrasound was found to be reliable, reproducible, and accurate for measuring the body fat of 94 obese adolescents [19]. The fat mass estimated from ultrasound correlated closely with DXA measurements in both females (*r* = 0.958, SEE = 2.9 kg) and males (*r* = 0.981, SEE = 2.5 kg). Additionally, a decrease in DXA-measured body fat of 13 adolescents following 6 months of treatment correlated closely with the decrease measured by ultrasound (*r* = 0.95). Bazzocchi et al. [44] came to a similar conclusion that ultrasound was reliable, reproducible, and accurate compared to CT in their sample of 26 nonobese and 29 obese patients, and Pereira et al. [53] recommended ultrasound as the preferred diagnostic method for assessing fat and lean mass in morbidly obese patients before and after bariatric surgery.

 The use of ultrasound to monitor the development of the fetus is well known; however, ultrasound can also be used to assess the health of the expecting mother. Bartha et al. [54] used ultrasound to measure the subcutaneous and visceral fat of 30 pregnant women at 11 to 14 weeks of gestation. They reported that the ultrasound-measured visceral fat correlated better with metabolic risk factors than pregestational BMI. Kinoshita and Itoh [55] used ultrasound to track the changes in the thicknesses of the preperitoneal and subcutaneous fat layers during pregnancy. They found a significant increase in the preperitoneal and preperitoneal/subcutaneous ratio during the third trimester compared to the first two trimesters. In a review of methods for determining maternal body composition, McCarthy et al. [56] acknowledged that ultrasound has been underutilized in assessing maternal fat stores despite its widespread use in obstetrics and gynecology. However, assessing maternal body composition may predict perinatal outcomes more accurately than maternal weight.

Another clinical population that presents special challenges for traditional body composition techniques is individuals with spinal cord injury. Due to reduced mobility, it is impractical and potentially unsafe to attempt certain body composition procedures, such as hydrostatic weighing, on this population. The portability, ease of use, and ability to measure regional composition makes ultrasound an attractive tool to assess the body composition of the spinal cord injured. Emmons et al. [57] took anthropometric, DXA, and ultrasound measurements on 24 spinal cord injured and 20 able-bodied men. Waist circumference and waist-to-hip ratio were correlated with visceral adiposity (*r* = 0.55) in the spinal cord injured group. The authors suggested that ultrasound may be a useful tool in the assessment of cardiometabolic disorders of the disabled.

 Ultrasound can also be used in clinical conditions that involve muscle wasting or abnormal fat distribution patterns. For example, Campbell and colleagues [58] noted that muscle wasting is often difficult to monitor during illness because of abnormal fluid retention that often affects the accuracy of many body composition methods. These authors used ultrasound to measure muscle thickness of the biceps, anterior forearm, and anterior thigh as a means to monitor muscle wasting in patients with multiple organ failure. Ultrasound has also been used to study the effect of antiretroviral drugs on visceral fat [59] and adipose redistribution [21] in HIV-infected patients. A side effect of antiretroviral therapy for HIV is a lipodystrophy syndrome known as HARS (HIV-associated adipose redistribution syndrome) which is characterized by fat being distributed disproportionally on the dorsocervical region (“buffalo hump”), an increase in intra-abdominal fat, and wasting of subcutaneous fat in the extremities and face [60]. Gulizia et al. [21] reported low intraobserver variability and good interobserver reliability when using ultrasound to assess body fat changes related to HARS. However, these researchers noted that training and practice improves interobserver agreement. Guimarães and colleagues [59] found that HIV patients on antiretroviral therapy had increased visceral adipose thickness and cardiometabolic risk factors compared to those not on the treatment.

## 7. New Technology

 Previous ultrasound research used B-mode ultrasound designed for diagnostic imaging at varying frequencies, but typically about 5 MHz, in order to obtain an image of subcutaneous fat. However, there are several emerging A-mode ultrasound devices of interest to body composition researchers and clinicians. Pineau et al. [18] described an A-mode ultrasound using a 2.25 MHz linear array probe (US Box, Lecoeur Electronique Co., Chuelles, France). Using intra-abdominal and midthigh measurements, they developed a model to estimate fat mass with DXA as the reference method. The ultrasound estimates of body fat percentage (*r* ^2^ = 0.96, SEE = 2.03, and TE = 1.00) were superior to estimates from BIA (*r* ^2^ = 0.85, SEE = 4.38, and TE = 2.57) and air displacement plethysmography (*r* ^2^ = 0.88, SEE = 3.68, and TE = 2.99). Subsequently, this research team validated this device for use on obese adolescents [19] and recently recalibrated it with a more conventional DXA [61].

**Figure 4:**
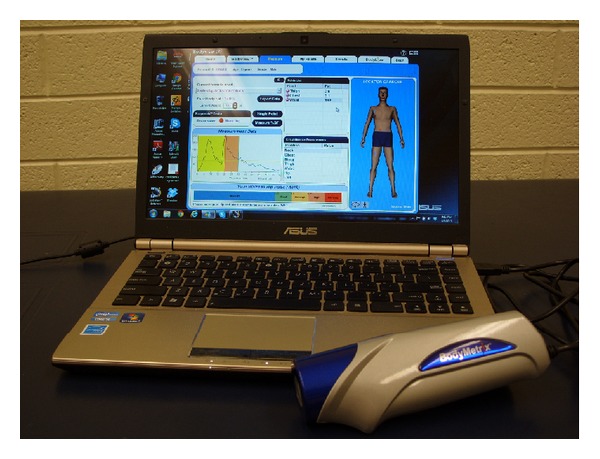
BodyMetrix ultrasound with body composition software.

**Figure 5:**
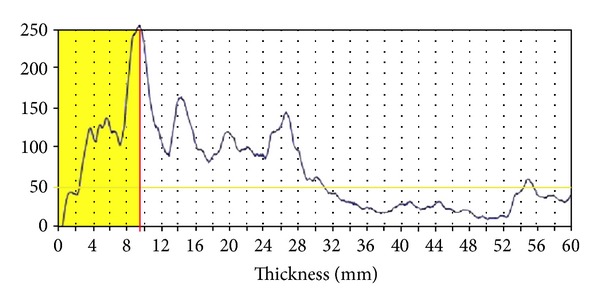
An A-mode ultrasound graph of an abdominal scan. Signal amplitude is on the *y*-axis and tissue depth is on the *x*-axis. The shaded area represents the subcutaneous fat, in this case about 9.5 mm.

A few years ago, a small, portable, hand-held 2.5 MHz A-mode ultrasound transducer designed specifically for the purpose of body composition assessment (BodyMetrix, BX2000, IntelaMetrix, Inc., Livermore, CA) arrived on the market. The ultrasound wand connects to a laptop computer via a USB cable (Figure 4). Proprietary software (Body View, IntelaMetrix, Inc., Livermore, CA) creates a measurement graph with tissue thickness or depth on the horizontal axis and the reflected ultrasound signal on the vertical axis (Figure 5). The software assumes the acoustic reflections of the fat-muscle and muscle bone interfaces to be 0.012 and 0.22, respectively [16]. The software will also calculate total body fat from the ultrasound measurements of standardized sites, such as the skinfold sites described by Jackson and Pollock [62], thereby creating a user-friendly method to estimate total body fat percentage from ultrasound. Additionally, image scans can be obtained (Figure 1) if tissue thickness is of greater interest than an estimate of body fat percentage.

There is a paucity of research available on this ultrasound system designed specifically for body composition assessment. In research presented at a 2006 conference but not published, Lyon et al. [63] reported high intraclass correlations (ICC) between the BodyMetrix BX2000 and skinfolds in young, lean, athletic males (*n* = 15) and females (*n* = 24). The ICCs for the sum of 7 skinfolds were 0.942 and 0.991 for women and men, respectively. There was also good agreement between the estimate of body fat percentage for women (skinfold = 18.7 ± 3.6% BF; ultrasound = 18.4 ± 3.7% BF) and men (skinfold = 10.7 ± 4.2% BF; ultrasound = 10.2 ± 3.9% BF).

Utter and Hager [64] compared fat-free mass (FFM) estimates from the BodyMetrix BX2000, skinfolds, and hydrostatic weighing in 70 high school wrestlers. The ultrasound estimate of FFM was significantly (*P* < 0.001) correlated with the estimate from hydrostatic weighing (*r* = 0.97). Furthermore, there was better agreement between ultrasound (57.2 ± 9.7 kg) and hydrostatic weighing (57.0 ± 9.9 kg) than between skinfolds (54.9 ± 8.8 kg) and hydrostatic weighing, as well as a lower SEE for ultrasound (2.40 kg) than skinfolds (2.74 kg). The authors concluded that the BodyMetrix BX2000 provided an acceptable estimate of FFM for high school wrestlers. 

In contrast to these studies, Ulbricht et al. [65] were more critical of this ultrasound unit when it was recently tested on a group of 30 overweight (BMI > 25 kg/m^2^) and 30 normal weight Brazilian military. They reported weak, nonsignificant correlations between skinfolds and ultrasound at the majority of anatomical locations tested. Nevertheless, there was good agreement and no significant difference in the total body fat percentage estimated from skinfolds (13.25 ± 6.32%) and ultrasound (12.73 ± 5.95%). 

It appears that the BodyMetrix BX2000 with Body View software could be the user-friendly ultrasound alternative to skinfolds and other field methods for estimating body fat percentage. This is a breakthrough in moving ultrasound from just providing accurate tissue thickness images at a specific location to using ultrasound to estimate total body fat. However, with only three known studies [63–65], including one with weak correlations at individual measurement sites [65], more validity and reliability studies are needed.

## 8. Summary 

 Despite 5 decades since the first published account of ultrasound being used to measure adipose tissue, this technology is often forgotten or ignored by body composition clinicians and researchers. However, there is substantial evidence that it is a reliable, reproducible, accurate, fast, and safe method to measure subcutaneous and visceral fat as well as muscle thickness. The fact that it is a small, portable, and relatively inexpensive imaging device that does not involve radiation gives it many advantages over other imaging devices and laboratory body composition techniques. Additionally, the ability to assess regional composition provides another advantage over many other methods and allows for unique assessments of some clinical populations. However, the lack of standardized procedures and results being highly dependent on the skill of the operator are limitations to ultrasound being used as a body composition technique. New, user-friendly devices with accompanying software designed specifically for body composition analysis may help to minimize these limitations, but they have not yet been adequately validated.

## Figures and Tables

**Table 1:** Advantages and limitations of using ultrasound for assessing body fat.

Advantages	Limitations
(1) Lower cost than laboratory methods	(1) Higher cost than field methods
(2) High accuracy and precision in the hands of an experienced technician	(2) Requires experienced technician, considerable skill is necessary
(3) Capable of regional and segmental measurements	(3) Measurement procedures and techniques are not yet standardized
(4) Minimal tissue compression	(4) Inherent artifacts (fascia etc.)
(5) Noninvasive and no ionizing radiation	
(6) Applicable for testing in the field	
(7) Can measure other tissue thicknesses (muscle and bone)	
(8) Short testing time, rapid procedure	
